# Ammonia-Induced Brain Edema Requires Macrophage and T Cell Expression of Toll-Like Receptor 9

**DOI:** 10.1016/j.jcmgh.2019.08.002

**Published:** 2019-08-08

**Authors:** Godhev Kumar Manakkat Vijay, Changyun Hu, Jian Peng, Irma Garcia-Martinez, Rafaz Hoque, Rejina Mariam Verghis, Yun Ma, Wajahat Zafar Mehal, Debbie Lindsay Shawcross, Li Wen

**Affiliations:** 1 Liver Sciences Department, Faculty of Life Sciences and Medicine, King’s College London, London, United Kingdom; 2 Section of Endocrinology, Department of Internal Medicine, Yale University School of Medicine, New Haven, Connecticut; 3 Section of Digestive Diseases, Department of Internal Medicine, Yale University School of Medicine, New Haven, Connecticut; 4 Welcome Wolfson Institute of Experimental Medicine, School of Medicine, Dentistry and Biomedical Science, Queens University, Belfast, United Kingdom

**Keywords:** Ammonia, Brain edema, Immune dysfunction, Toll-like receptor 9, ALF, acute liver failure, BW, brain water, IFNγ, interferon gamma, IL, interleukin, mAbs, monocloncal antibodies, Na-Ac, sodium acetate, NH_4_-Ac, ammonium acetate, TNFα, tumor necrosis factor alpha, TLR9, Toll-like receptor 9, WT, wild-type

## Abstract

**Background & Aim:**

Ammonia is central in the pathogenesis of brain edema in acute liver failure (ALF) with infection and systemic inflammation expediting development of intracranial hypertension (ICH). Patients with acetaminophen-induced ALF have increased neutrophil TLR9 expression which can be induced by ammonia. We determined whether ammonia-induced brain edema and immune dysfunction are mediated by TLR9 and if this could be prevented in a TLR9-deficient mouse model.

**Methods:**

Ammonium acetate (NH_4_-Ac; 4mmol/kg) was injected intraperitoneally in wild type (WT), *Tlr9*^*-/-*^ and *Lysm-Cre Tlr9*^*fl/fl*^ mice (TLR9 absent in neutrophils and macrophages including Kupffer cells) and compared to controls. Six hours after NH_4_-Ac injection, intracellular cytokine production was determined in splenic macrophages, CD4^+^ and CD8^+^ T cells. Brain water (BW) and total plasma DNA (tDNA) were also measured. The impact of the TLR9 antagonist ODN2088 (50μg/mouse) was evaluated.

**Results:**

Following NH_4_-Ac injection, BW, macrophage and T cell cytokine production increased (*P* < .0001) in WT but not *Tlr9*^*-/-*^ mice (*P* < .001). ODN2088 inhibited macrophage and T cell cytokine production (*P* < .05) and prevented an increase in BW (*P* < .0001). Following NH_4_-Ac injection, macrophage cytokine production and BW were ameliorated in *Lysm-Cre Tlr9*^*fl/fl*^ mice compared to WT mice (*P* < .05) but there was no difference compared to *Tlr9*^*-/-*^ mice. Following NH_4_-Ac injection, plasma tDNA levels increased in WT and *Tlr9*^*-/-*^ mice (*P* < .05) suggesting that TLR9 may be activated by DNA released from ammonia-stimulated cells.

**Conclusion:**

Ammonia-induced brain edema requires macrophage and T cell expression of TLR9**.** Amelioration of brain edema and lymphocyte cytokine production by ODN2088 supports exploration of TLR9 antagonism in early ALF to prevent progression to ICH.

## Graphical abstract

**Figure:**
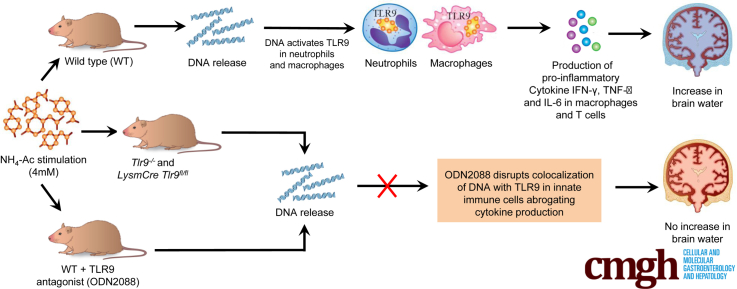

See editorial on page 649.

**Summary**

Utilizing a TLR9-deficient mouse model and the TLR9 antagonist ODN2088, we have shown that ammonia-induced brain edema requires macrophage and T cell expression of TLR9 supporting exploration of TLR9 antagonism in acute liver failure.

Ammonia plays a pivotal role in the development of hepatic encephalopathy and brain edema in acute liver failure (ALF).^1^, ^2^, ^3^ A robust systemic inflammatory response and susceptibility to developing infection are common in ALF exacerbate the development of ammonia-induced brain edema and are major prognosticators.^4^, ^5^, ^6^, ^7^, ^8^, ^9^, ^10^ Experimental models have unequivocally associated ammonia exposure with astrocyte swelling and brain edema, potentiated by proinflammatory cytokines.^11^, ^12^, ^13^, ^14^

Toll-like receptor 9 (TLR9) is an innate pattern recognition receptor that binds to the CpG motif of bacterial and mammalian DNA.^15^ TLR9 plays an important role in acetaminophen-induced liver inflammation,^16^ and we recently reported that ammonia along with DNA induces neutrophil TLR9 expression in patients with acetaminophen-induced ALF and advanced hepatic encephalopathy.^17^ Although the evidence base supporting the relationship between ammonia, inflammation and brain edema is robust in ALF, there is a paucity of data characterizing the specific pathogenic mechanisms entailed. We hypothesized that ammonia-induced brain edema and immune dysfunction are mediated by TLR9. As TLR9 is necessary for the development of acetaminophen-induced acute liver injury in murine models,^16^ the hypothesis could only be tested in a murine model exposed to ammonium acetate (NH_4_-Ac) without liver injury.

Using an acute hyperammonemic mouse model, we demonstrated that ammonia-induced brain edema and immune dysfunction, as measured by increased brain water (BW) content and intracellular cytokine production of macrophages and T cells are mediated through TLR9. In mice, hyperammonemia resulted in DNA release and activation of TLR9 inducing downstream inflammatory cytokine production. TLR9 in lysozyme expressing cells was critical for the development of brain edema and immune dysfunction. Administration of a TLR9 antagonist abrogated inflammation and prevented brain edema.

## Results

### Ammonia-Induced Brain Edema and Changes in the Liver Were Dependent on TLR9

**Figure 1:**
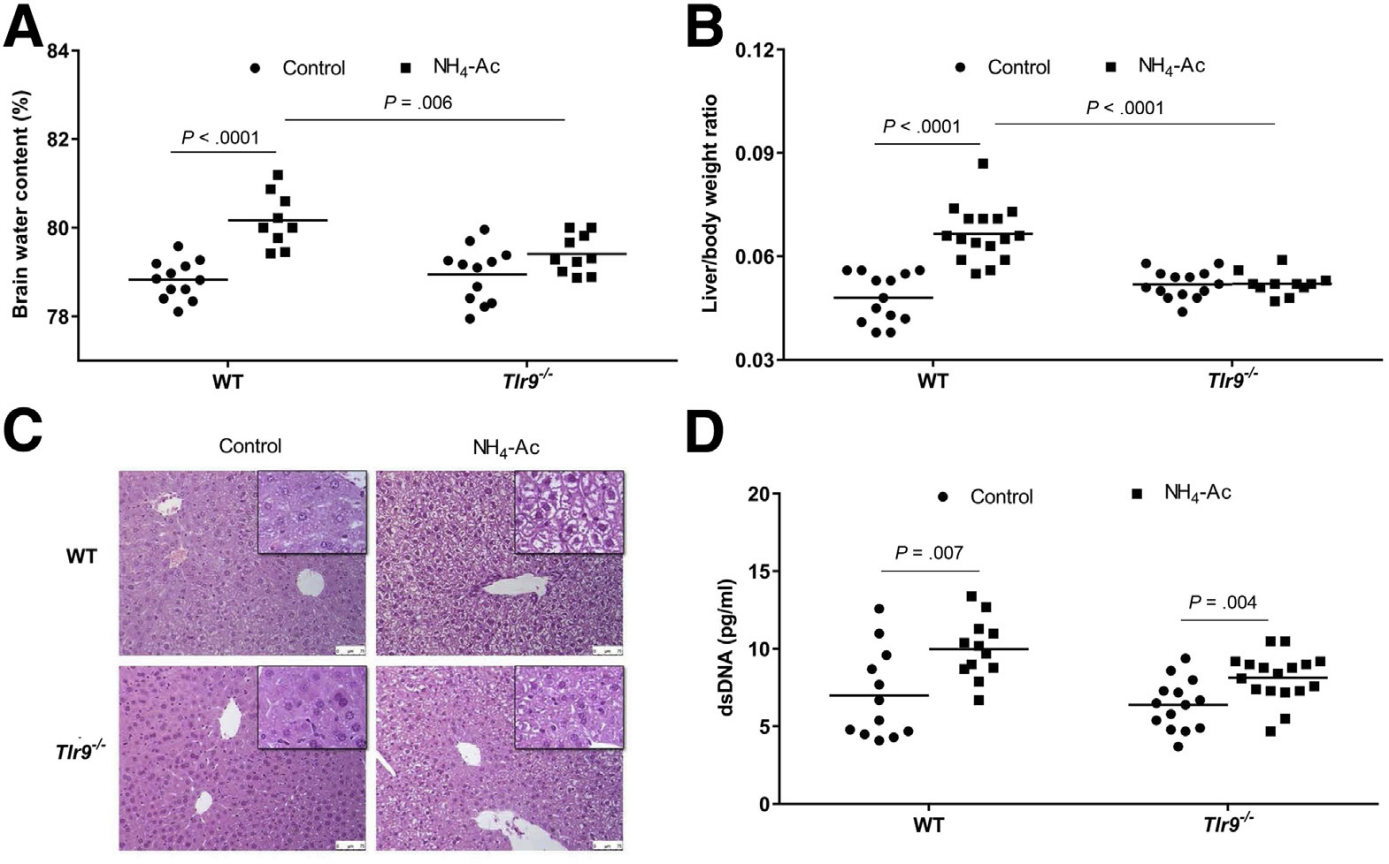
**TLR9 stimulates ammonia-induced brain edema and liver-to-body weight ratio through DNA.** (*A*) Following NH_4_-Ac stimulation, there was a significant increase in the BW content in WT mice (n = 9) compared with control mice (n = 12) (*P <* .0001) (mean difference: 1.35; 95% confidence interval [CI], 0.9 to 1.8), which was ameliorated in *Tlr9*^*–/–*^ mice (n = 10) (*P =* .006) (mean difference: –0.76; 95% CI, –1.3 to –0.25). (*B*) Following NH_4_-Ac stimulation, there was a significant increase in the liver-to-body weight ratio in WT mice (n = 16) compared with control mice (n = 13) (*P <* .0001) (mean difference: 0.019; 95% CI, 0.013 to 0.024), which was ameliorated in *Tlr9*^*–/–*^ mice (n = 11) (*P <* .0001) (mean difference: –0.014; 95% CI, –0.019 to –0.009). (*C*) Following NH_4_-Ac stimulation, there was a significant increase in the hepatocyte swelling in the liver histology (hematoxylin and eosin stain, 200× magnification) in WT mice compared with control mice, where the cytoplasm of cells remained intact. Following NH_4_-Ac stimulation, there was no difference in the hepatocyte morphology in *Tlr9*^*–/–*^ mice compared with control mice but the hepatocyte swelling was reduced compared with WT mice. The images in the black box inset are the representative images at 400× magnification. (*D*) Following NH_4_-Ac stimulation, there was a significant increase in the total plasma DNA level in WT mice (n = 12) compared with control mice (n = 12) (*P =* .007) (mean difference: 2.97; 95% CI, 0.88 to 5) and in *Tlr9*^*–/–*^ mice (n = 17) compared with control mice (n = 14) (*P =* .004) (mean difference: 1.76; 95% CI, 0.6 to 2.9).

To determine whether TLR9 plays a role in ammonia-induced brain edema, we evaluated the BW content of wild-type (WT) and *Tlr9*^*–/–*^ mice 6 hours after a single dose of NH_4_-Ac (4 mM) injection (intraperitoneal). Following NH_4_-Ac stimulation, there was a significant increase in the BW content in WT mice compared with control mice; which was significantly decreased in *Tlr9*^*–/–*^ mice compared with WT mice (Figure 1*A*) indicating that TLR9 plays an important role in the development of brain edema. To determine whether the increased BW content was associated with any changes in the liver, we assessed the liver-to-body weight ratio and liver histopathology after NH_4_-Ac stimulation. In WT mice, there was a significant increase in liver-to-body weight ratio with evidence of hepatocyte swelling but not necrosis (there was no rise in serum aspartate and alanine transaminases), a finding which was abrogated in *Tlr9*^*–/–*^ mice (Figure 1*B* and *C*). We hypothesized that DNA released following NH_4_-Ac stimulation can bind to TLR9 resulting in activation of the innate immune system. To test our hypothesis, we measured plasma DNA. Total plasma dsDNA levels were significantly increased in WT mice and *Tlr9*^*–/–*^ mice following NH_4_-Ac stimulation compared with control mice, but there was no difference in *Tlr9*^*–/–*^ mice compared with WT mice following NH_4_-Ac stimulation (Figure 1*D*).

### Ammonia Altered the Function of Macrophages and T Cells in a TLR9-Dependent Manner

**Figure 2:**
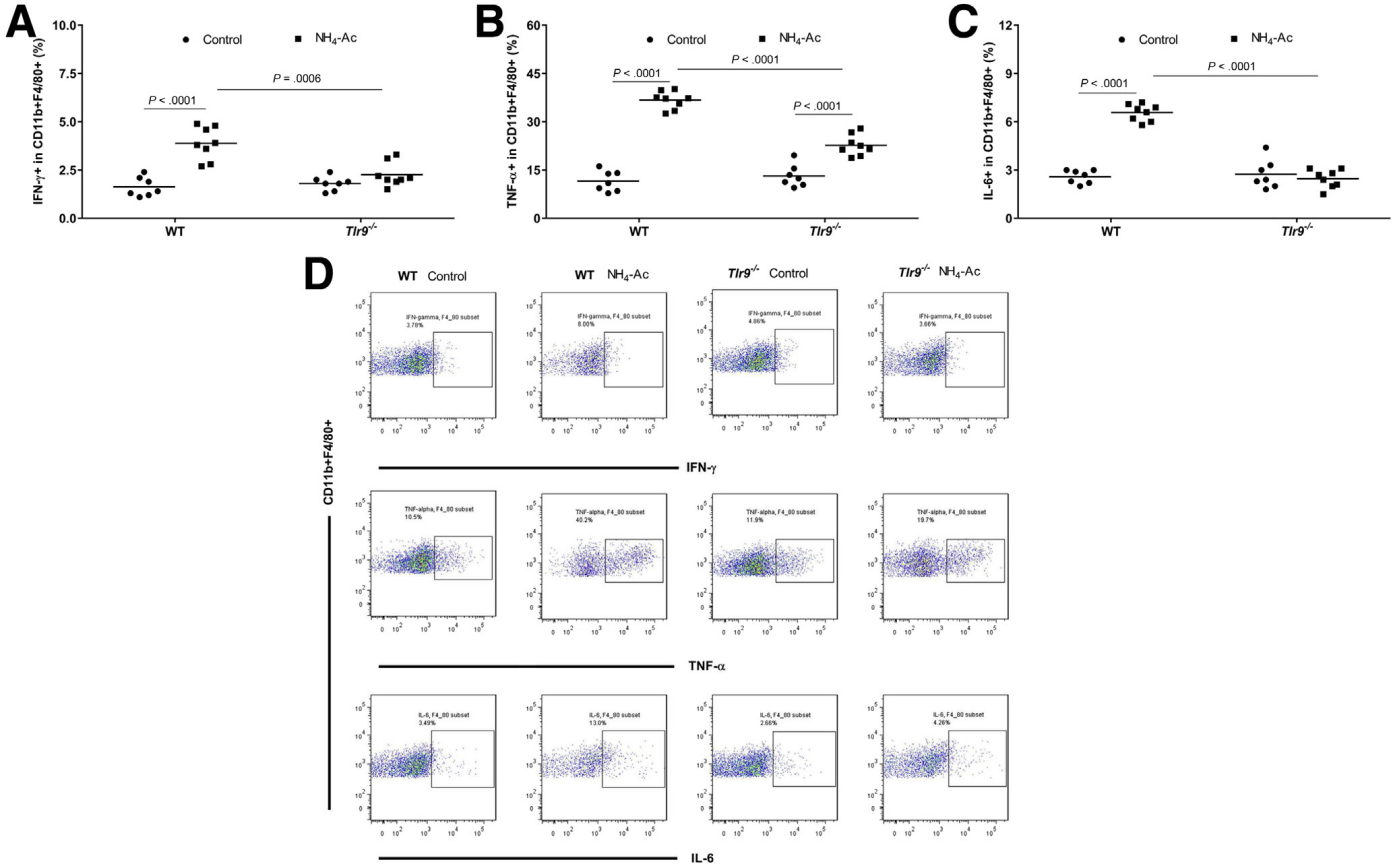
**Ammonia-induced intracellular cytokine production by macrophages is mediated by TLR9.** (*A*) Following NH_4_-Ac stimulation, there was a significant increase in the intracellular cytokine IFNγ produced by splenic macrophages in WT mice (n = 8) compared with control mice (n = 7) (*P <* .0001) (mean difference: 2.3; 95% CI, 1.5 to 3), which was ameliorated in *Tlr9*^*–/–*^ mice (n = 8) (*P =* .0006) (mean difference: –1.6; 95% CI, –2.4 to –0.83). (*B*) Following NH_4_-Ac stimulation, there was a significant increase in the intracellular cytokine TNFα produced by splenic macrophages in WT mice (n = 8) compared with control mice (n = 7) (*P <* .0001) (mean difference: 25; 95% CI, 22 to 28.5), which was ameliorated in *Tlr9*^*–/–*^ mice (n = 8) (*P <* .0001) (mean difference: –14; 95% CI, –17.2 to –10.8). (*C*) Following NH_4_-Ac stimulation, there was a significant increase in the intracellular cytokine IL-6 produced by splenic macrophages in WT mice (n = 8) compared with control mice (n = 7) (*P <* .0001) (mean difference: 4; 95% CI, 3.5 to 4.5), which was ameliorated in *Tlr9*^*–/–*^ mice (n = 8) (*P <* .0001) (mean difference: –4; 95% CI, –4.7 to –3.5). (*D*) Representative FACS plots of intracellular cytokines IFNγ, TNFα, and IL-6 produced by splenic macrophages in WT mice and *Tlr9*^*–/–*^ control mice, and following NH_4_-Ac stimulation.

**Figure 3:**
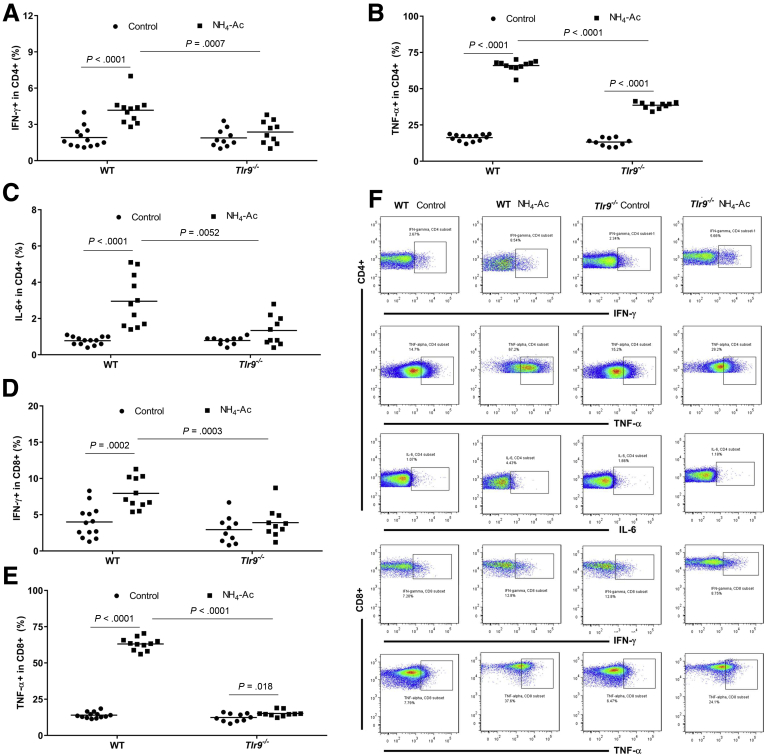
**Ammonia-induced intracellular cytokine production by T cells is mediated by TLR9.** (*A*) Following NH_4_-Ac stimulation, there was a significant increase in the intracellular cytokine IFNγ produced by splenic CD4^+^ T cells in WT mice compared with control mice (*P <* .0001) (median difference: 2.7; 95% CI, 1.5 to 3), which was ameliorated in *Tlr9*^*–/–*^ mice compared with WT mice (*P =* .0007) (median difference: –1.9; 95% CI, –2.8 to –0.8). (*B*) Following NH_4_-Ac stimulation, there was a significant increase in the intracellular cytokine TNFα produced by splenic CD4^+^ T cells in WT mice compared with control mice (*P <* .0001) (mean difference: 49.5; 95% CI, 47 to 52), which was ameliorated in *Tlr9*^*–/–*^ mice compared with WT mice (*P <* .0001) (mean difference: –27.5; 95% CI, –30 to –25). (*C*) Following NH_4_-Ac stimulation, there was a significant increase in the intracellular cytokine IL-6 produced by splenic CD4^+^ T cells in WT mice compared with control mice (*P <* .0001) (mean difference: 2.2; 95% CI, 1.4 to 3), which was ameliorated in *Tlr9*^*–/–*^ mice compared with WT mice (*P =* .0052) (mean difference: –1.6; 95% CI, –2.7 to –0.5). (*D*) Following NH_4_-Ac stimulation, there was a significant increase in the intracellular cytokine IFNγ produced by splenic CD8^+^ T cells in WT mice compared with control mice (*P =* .0002) (mean difference: 3.9; 95% CI, 2.1 to 5.8), which was ameliorated in *Tlr9*^*–/–*^ mice compared with WT mice (*P =* .0003) (median difference: –3.5; 95% CI, –6.4 to –2.1). (*E*) Following NH_4_-Ac stimulation, there was a significant increase in the intracellular cytokine TNFα produced by splenic CD8^+^ T cells in WT mice compared with control mice (*P <* .0001) (mean difference: 49; 95% CI, 46 to 52), which was ameliorated in *Tlr9*^*–/–*^ mice compared with WT mice (*P <* .0001) (mean difference: –48; 95% CI, –51 to –45). (*F*) Representative FACS plots of the intracellular cytokines IFNγ, TNFα, and IL-6 produced by splenic T cells in WT mice and *Tlr9*^*–/–*^ control mice and following NH_4_-Ac stimulation. WT control mice (n = 11) and NH_4_-Ac-treated mice (n = 13); *Tlr9*^*–/–*^ control mice (n = 10) and NH_4_-Ac-treated mice (n = 10).

**Figure 4:**
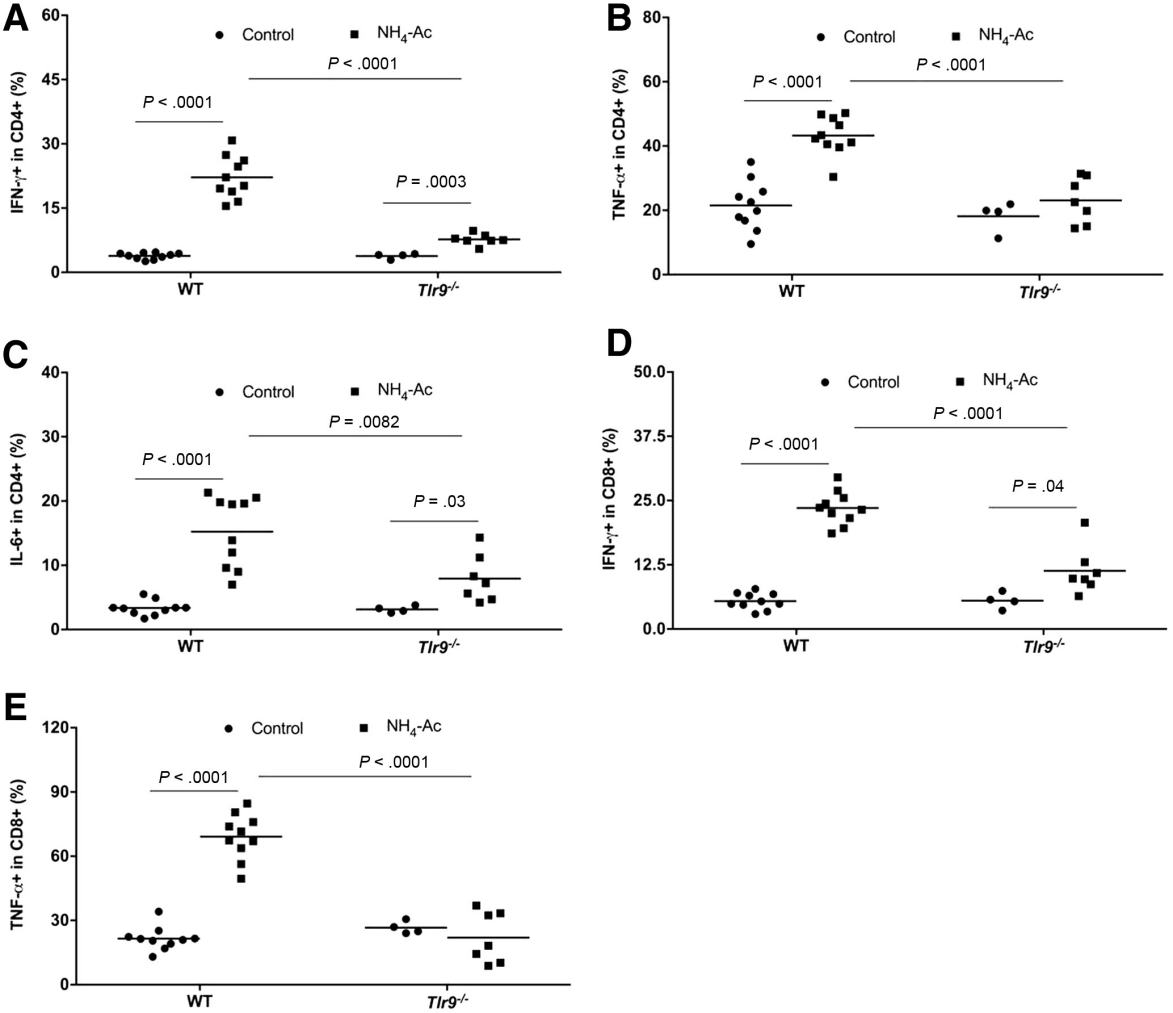
**Ammonia-induced intracellular cytokine production by liver-infiltrated T cells.** (*A*) Following NH_4_-Ac stimulation, there was a significant increase in the intracellular cytokine IFNγ produced by liver-infiltrated CD4^+^ T cells in WT mice compared with control mice (*P <* .0001) (median difference: 18.3; 95% CI, 15 to 21.6), which was ameliorated in *Tlr9*^*–/–*^ mice compared with WT mice (*P <* .0001) (median difference: –14.5; 95% CI, –18.6 to –10.3). (*B*) Following NH_4_-Ac stimulation, there was a significant increase in the intracellular cytokine TNFα produced by liver-infiltrated CD4^+^ T cells in WT mice compared with control mice (*P <* .0001) (mean difference: 21.4; 95% CI, 14.5 to 28.2), which was ameliorated in *Tlr9*^*–/–*^ mice compared with WT mice (*P <* .0001) (mean difference: –20; 95% CI, –27 to –12.7). (*C*) Following NH_4_-Ac stimulation, there was a significant increase in the intracellular cytokine IL-6 produced by liver-infiltrated CD4^+^ T cells in WT mice compared with control mice (*P <* .0001) (mean difference: 11.9; 95% CI, 8.1 to 15.6), which was ameliorated in *Tlr9*^*–/–*^ mice compared with WT mice (*P =* .0082) (mean difference: –7.3; 95% CI, –12.4 to –2.2). (*D*) Following NH_4_-Ac stimulation, there was a significant increase in the intracellular cytokine IFNγ produced by liver-infiltrated CD8^+^ T cells in WT mice compared with control mice (*P <* .0001) (mean difference: 18.3; 95% CI, 15.8 to 20.9), which was ameliorated in *Tlr9*^*–/–*^ mice compared with WT mice (*P <* .0001) (median difference: –12.4; 95% CI, –16.7 to –8.2). (*E*) Following NH_4_-Ac stimulation, there was a significant increase in the intracellular cytokine TNFα produced by liver-infiltrated CD8^+^ T cells in WT mice compared with control mice (*P <* .0001) (mean difference: 47.3; 95% CI, 39 to 56), which was ameliorated in *Tlr9*^*–/–*^ mice compared with WT- mice (*P <* .0001) (mean difference: –44; 95% CI, –56 to –32). WT control mice (n = 10) and NH_4_-Ac-treated mice (n = 10); *Tlr9*^*–/–*^ control mice (n = 4) and NH_4_-Ac-treated mice (n = 7).

**Figure 5:**
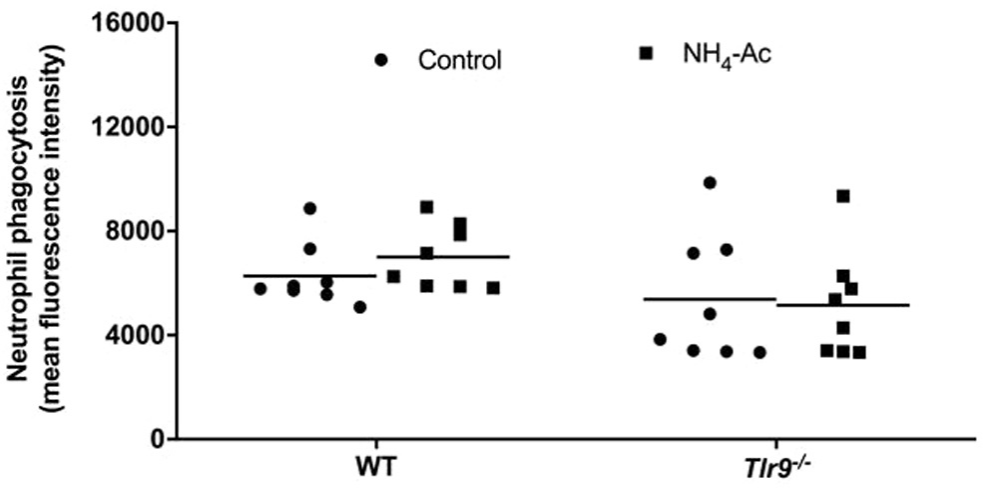
**Neutrophil phagocytosis measured by dextran fluorescein isothiocyanate (FITC) using flow cytometry in WT and Tlr9**^**–/–**^**mice after NH**_**4**_**-Ac stimulation.** Following NH_4_-Ac stimulation, there was no difference in the mean fluorescence intensity of dextran FITC in neutrophils isolated from whole blood in WT mice or *Tlr9*^*–/–*^ mice compared with control mice.

To determine whether systemic inflammation contributed to the ammonia-induced increase in BW content through a TLR9-mediated pathway, we measured intracellular cytokine (interferon gamma [IFNγ], tumor necrosis factor alpha [TNFα], and interleukin [IL]-6) production in T cells and macrophages isolated from spleen and liver in WT mice and *Tlr9*^*–/–*^ mice following NH_4_-Ac stimulation. There was a significant increase in the intracellular cytokines produced by macrophages (Figure 2*A–D*) and CD4^+^ and CD8^+^ T cells (Figure 3*A–F*) isolated from the spleen in WT mice following NH_4_-Ac stimulation, compared with control mice which was abrogated in *Tlr9*^*–/–*^ mice. A similar trend was observed in the immune T cells isolated from the liver (Figure 4). Neutrophil phagocytic activity was unaltered following NH_4_-Ac stimulation (Figure 5).

### Ammonia-Induced Brain Edema and Inflammation Were Independent of Acetate or pH

**Figure 6:**
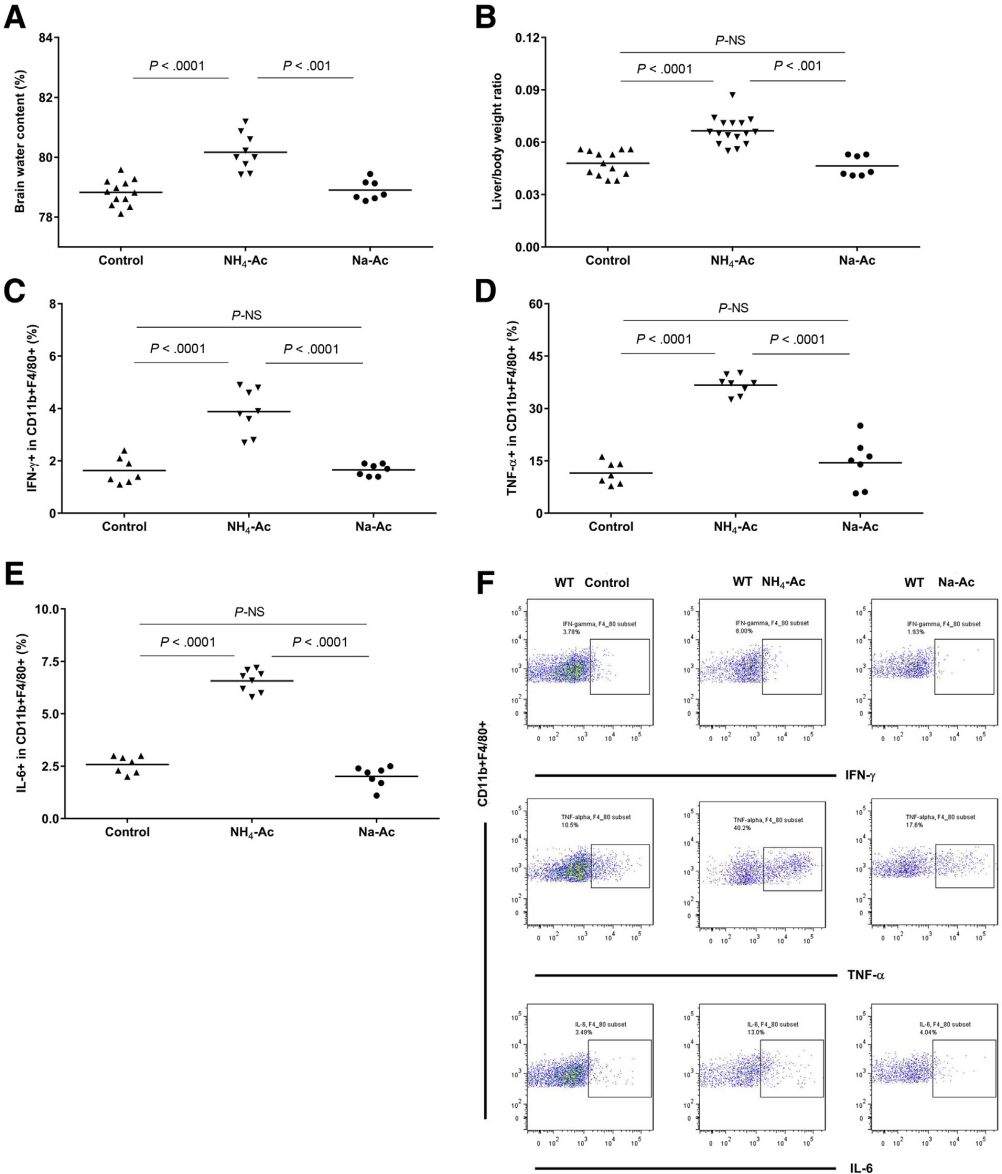
**Unaltered brain edema and intracellular cytokine production by macrophages after Na-Ac stimulation.** (*A*) Following Na-Ac stimulation, BW content remained unaltered in WT mice (n = 7) compared with control mice (n = 12), but was significantly reduced compared with the NH_4_-Ac–stimulated WT mice (n = 9) (*P <* .001) (mean difference: –1.1; 95% CI, –1.7 to –0.47). (*B*) Following Na-Ac stimulation, liver-to-body weight ratio remained unaltered in WT mice (n = 7) compared with control mice (n = 13), but was significantly reduced compared with the NH_4_-Ac–stimulated WT mice (n = 16) (*P <* .001) (mean difference: –0.02; 95% CI, –0.028 to –0.012). Following Na-Ac stimulation, the intracellular cytokine (*C*) IFNγ (*P <* .0001) (mean difference: –2.2; 95% CI, –3 to –1.4), (*D*) TNFα (*P <* .0001) (mean difference: –22.3; 95% CI, –28.3 to –16.3), and (*E*) IL-6 (*P <* .0001) (mean difference: –4.5; 95% CI, –5.2 to –3.9) produced by splenic macrophages were significantly reduced in WT mice (n = 7) compared with the NH_4_-Ac–stimulated WT mice (n = 8) but remained unaltered compared with control mice (n = 7). (*F*) Representative FACS plots of the intracellular cytokines IFNγ, TNFα, and IL-6 produced by splenic macrophages in WT control mice, NH_4_-Ac–stimulated WT mice and Na-Ac–stimulated WT mice.

Acetate has been reported to influence inflammation in acute alcoholic hepatitis^18^ and the increased toxicity of ammonium salts promotes ammonium gas transfer across the blood brain barrier due to the rise in blood pH and the direct effect of alkalinization.^19^ Therefore, to confirm whether the TLR9-mediated brain edema and inflammation were solely induced by ammonia and not by the acetate or changes in pH, an alternate salt of acetate, sodium acetate (NaCH_3_CO_2_) (Na-Ac) was injected in WT mice after adjusting for pH (same as NH_4_-Ac). Na-Ac (4 mM) did not alter the BW content, liver-to-body weight ratio, or intracellular cytokine production of various immune cell subsets in WT mice compared with control mice, unlike NH_4_-Ac (Figure 6*A–F*). These results confirm that the observed ammonia-induced cytokine production and brain edema were induced by ammonia per se, and not influenced by acetate or pH changes.

### Ammonia-Induced Brain Edema and Inflammation Were Mediated by TLR9 Expressed Within Lysozyme-Expressing Cells

**Figure 7:**
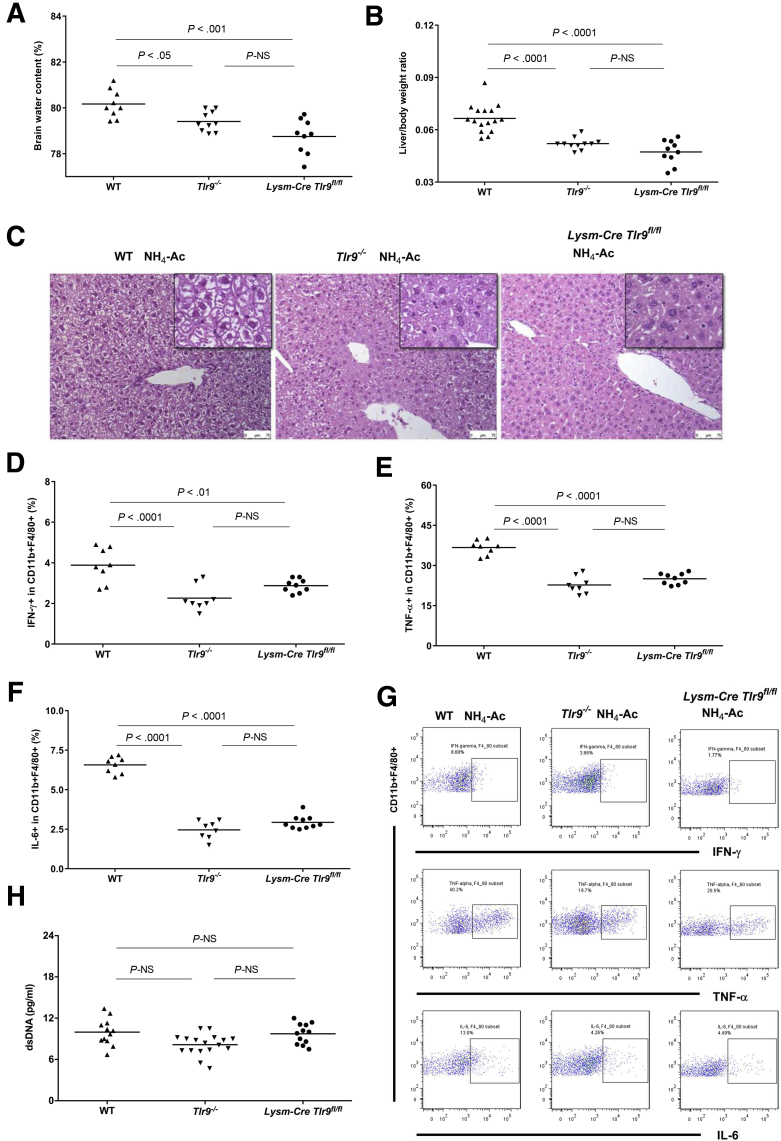
***TLR9 in lysosomal expressed cells stimulates ammonia-induced brain edema and cytokine production by macrophages.*** (*A*) Following NH_4_-Ac stimulation, BW content was significantly ameliorated in *Tlr9*^*–/–*^ mice (n = 10) (*P <* .05) (mean difference: –0.76; 95% CI, –1.5 to –0.06) and *Lysm-Cre Tlr9*^*fl/fl*^ mice (n = 9) (*P <* .001) (mean difference: –1.4; 95% CI, –2 to –0.7) compared with WT mice (n = 9). (*B*) Following NH_4_-Ac stimulation, liver-to-body weight ratio was significantly ameliorated in *Tlr9*^*–/–*^ mice (n = 11) (*P <* .0001) (mean difference: –0.015; 95% CI, –0.021 to –0.008) and *Lysm-Cre Tlr9*^*fl/fl*^ mice (n = 10) (*P <* .0001) (mean difference: –0.019; 95% CI, –0.026 to –0.013) compared with WT mice (n = 16). (*C*) Following NH_4_-Ac stimulation, there was a significant increase in the hepatocyte swelling in the liver histology (hematoxylin and eosin stain, 200× magnification) in WT mice compared with *Tlr9*^*–/–*^ mice and *Lysm-Cre Tlr9*^*fl/fl*^ mice, where the cytoplasm of cells remained intact. The images in the black box inset are the representative images at 400× magnification. (*D*) Following NH_4_-Ac stimulation, the intracellular cytokine IFNγ produced by splenic macrophages was significantly ameliorated in *Tlr9*^*–/–*^ mice (n = 8) (*P <* .0001) (mean difference: –1.6; 95% CI, –2.4 to –0.84) and *Lysm-Cre Tlr9*^*fl/fl*^ mice (n = 9) (*P <* .01) (mean difference: –1; 95% CI, –1.75 to –0.25) compared with WT mice (n = 8). (*E*) Following NH_4_-Ac stimulation, the intracellular cytokine TNFα produced by splenic macrophages was significantly ameliorated in *Tlr9*^*–/–*^ mice (n = 8) (*P <* .0001) (mean difference: –14; 95% CI, –17.4 to –10.6) and *Lysm-Cre Tlr9*^*fl/fl*^ mice (n = 9) (*P <* .0001) (mean difference: –11.7; 95% CI, –15 to –8.4) compared with WT mice (n = 8). (*F*) Following NH_4_-Ac stimulation, the intracellular cytokine IL-6 produced by splenic macrophages was significantly ameliorated in *Tlr9*^*–/–*^ mice (n = 8) (*P <* .0001) (mean difference: –4.1; 95% CI, –4.7 to –3.4) and *Lysm-Cre Tlr9*^*fl/fl*^ mice (n = 9) (*P <* .0001) (mean difference: –3.6; 95% CI, –4.2 to –3) compared with WT mice (n = 8). (*G*) Representative FACS plots of the intracellular cytokines IFNγ, TNFα, and IL-6 produced by splenic macrophages in WT mice, *Tlr9*^*–/–*^ mice and *Lysm-Cre Tlr9*^*fl/fl*^ mice following NH_4_-Ac stimulation. (*H*) There was no difference in the total plasma DNA level between WT mice (n = 12), *Tlr9*^*–/–*^ mice (n = 17) and *Lysm-Cre Tlr9*^*fl/fl*^ mice (n = 12).

**Figure 8:**
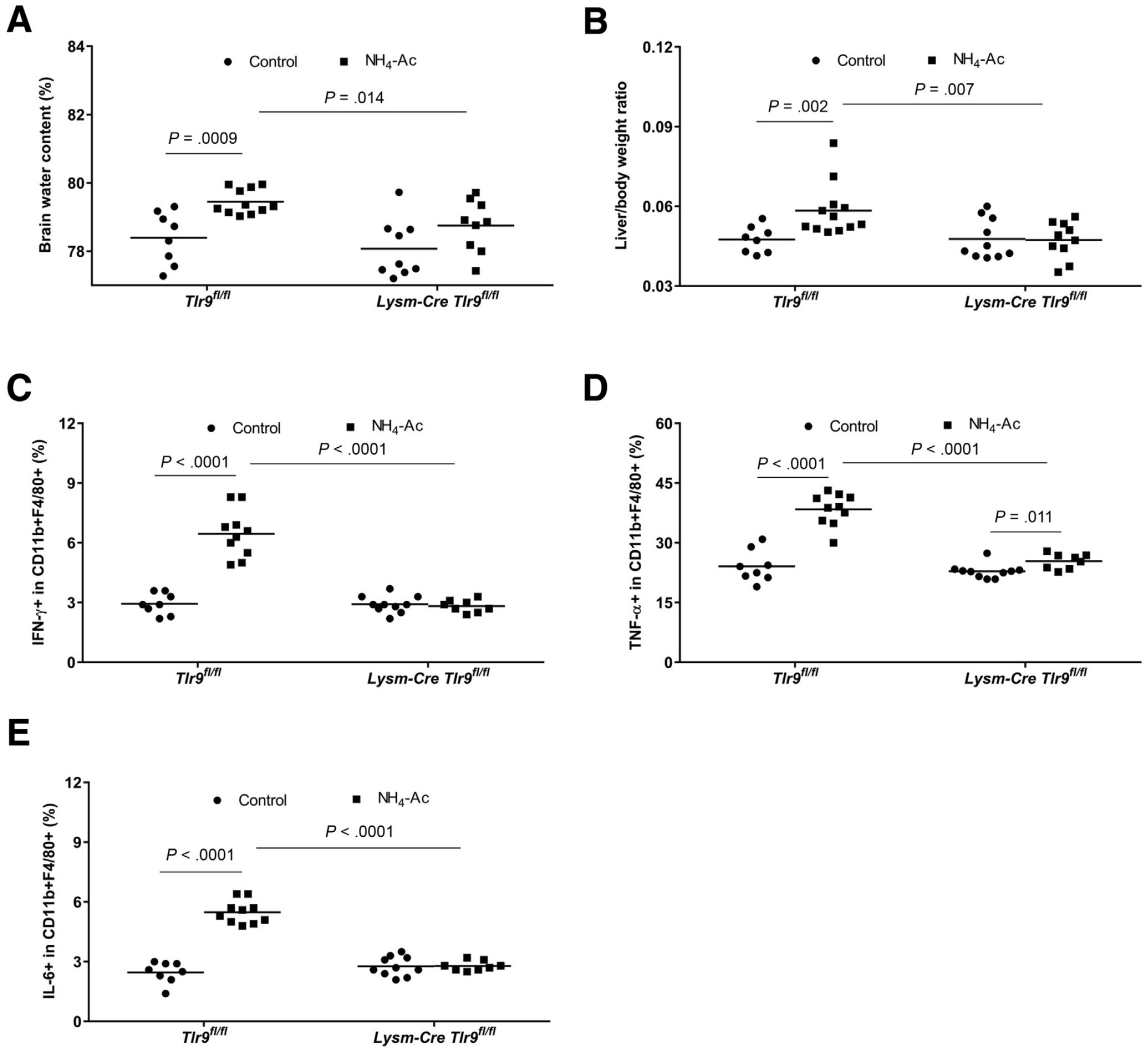
**Ammonia-induced brain edema and cytokine production in Lysm-Cre Tlr9**^**fl/fl**^**mice compared with Tlr9**^**fl/fl**^**mice.** (*A*) Following NH_4_-Ac stimulation, there was a significant increase in the BW content in *Tlr9*^*fl/fl*^ mice (n = 11) compared with control mice (n = 8) (*P =* .0009) (mean difference: 1.05; 95% CI, 0.5 to 1.6), which was ameliorated in *Lysm-Cre Tlr9*^*fl/fl*^ mice (n = 9) (*P =* .014) (mean difference: –0.7; 95% CI, –1.2 to –0.16). (*B*) Following NH_4_-Ac stimulation, there was a significant increase in the liver-to-body weight ratio in *Tlr9*^*fl/fl*^ mice (n = 12) compared with control mice (n = 8) (*P =* .002) (mean difference: 0.007; 95% CI, 0.003 to 0.015), which was ameliorated in *Lysm-Cre Tlr9*^*fl/fl*^ mice (n = 10) (*P =* .007) (mean difference: –0.008; 95% CI, –0.017 to –0.002). (*C*) Following NH_4_-Ac stimulation, there was a significant increase in the intracellular cytokine IFNγ produced by splenic macrophages in *Tlr9*^*fl/fl*^ mice (n = 10) compared with control mice (n = 8) (*P <* .0001) (mean difference: 3.5; 95% CI, 2.5 to 4.5), which was ameliorated in *Lysm-Cre Tlr9*^*fl/fl*^ mice (n = 8) (*P <* .0001) (mean difference: –3.6; 95% CI, –4.6 to –2.7). (*D*) Following NH_4_-Ac stimulation, there was a significant increase in the intracellular cytokine TNFα produced by splenic macrophages in *Tlr9*^*fl/fl*^ mice (n = 10) compared with control mice (n = 8) (*P <* .0001) (mean difference: 14.3; 95% CI, 10.2 to 18.3), which was ameliorated in *Lysm-Cre Tlr9*^*fl/fl*^ mice (n = 8) (*P <* .0001) (mean difference: –13; 95% CI, –16.3 to –9.7). (*E*) Following NH_4_-Ac stimulation, there was a significant increase in the intracellular cytokine IL-6 produced by splenic macrophages in *Tlr9*^*fl/fl*^ mice (n = 10) compared with control mice (n = 8) (*P <* .0001) (mean difference: 3; 95% CI, 2.5 to 3.5), which was ameliorated in *Lysm-Cre Tlr9*^*fl/fl*^ mice (n = 8) (*P <* .0001) (mean difference: –2.7; 95% CI, –3.2 to –2.2).

Our results showed that deletion of TLR9 tempered the proinflammatory state and abrogated the development of brain edema following ammonia stimulation. To examine the role of TLR9 in macrophages, we used *Lysm-Cre Tlr9*^*fl/fl*^ mice that have TLR9 specifically deleted in lysozyme-expressing cells, namely macrophages and neutrophils.^20^ NH_4_-Ac (4 mM) was therefore injected into *Lysm-Cre Tlr9*^*fl/fl*^ mice. Interestingly, we found that BW content, liver-to-body weight ratio, hepatocyte swelling, and cytokines produced by macrophages were significantly ameliorated in *Lysm-Cre Tlr9*^*fl/fl*^ mice compared with WT mice following NH_4_-Ac stimulation but with no difference compared with *Tlr9*^*–/–*^ mice (Figure 7*A–G*). There was no difference in the total plasma dsDNA levels in *Tlr9*^*–/–*^ mice and *Lysm-Cre Tlr9*^*fl/fl*^ mice compared with WT mice following NH_4_-Ac stimulation (Figure 7*H*). We also tested another control mouse strain, *Tlr9*^*fl/fl*^ mice (TLR9 is sufficient in all the cell types). As expected, *Tlr9*^*fl/fl*^ mice were not protected from NH_4_-Ac induced inflammation compared with *Lysm-Cre Tlr9*^*fl/fl*^ mice (Figure 8*A–E*).

### TLR9 Antagonism Abrogates Ammonia-Induced Brain Edema and Inflammation

**Figure 9:**
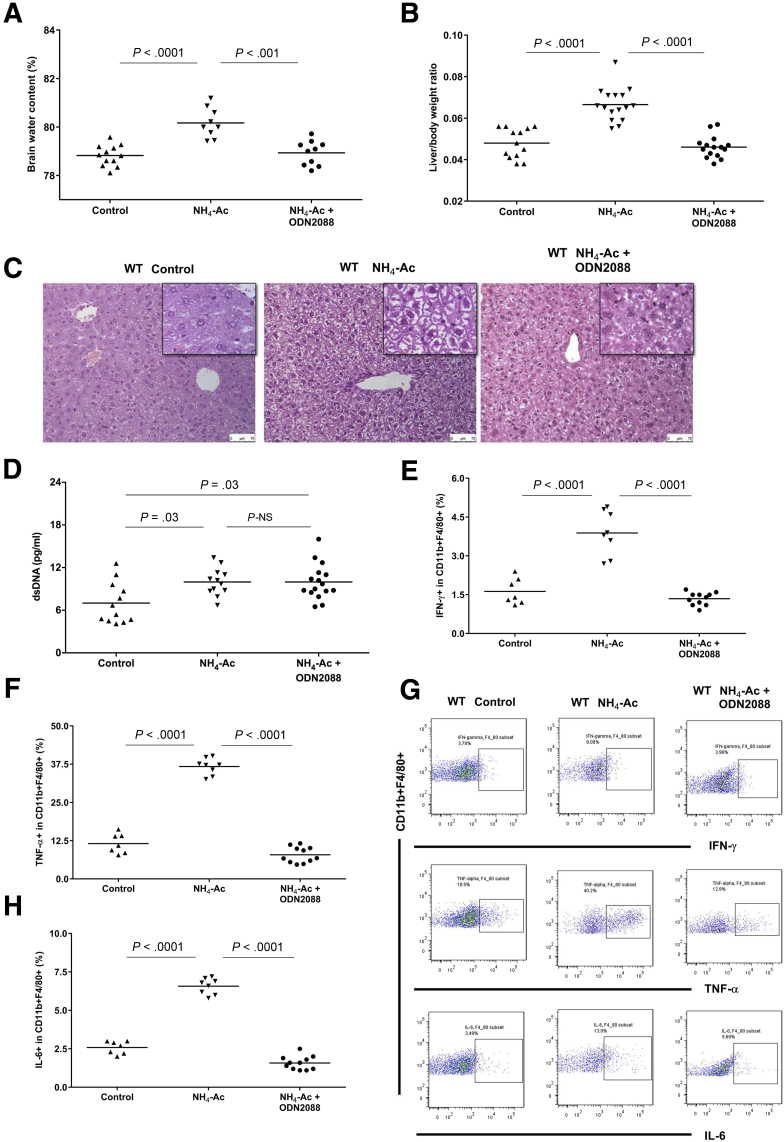
**ODN2088 inhibits the ammonia-induced brain edema and cytokine production by macrophages in WT mice.** (*A*) Administration of the TLR9 antagonist, ODN2088, along with NH_4_-Ac prevented the rise in BW content (n = 10) (*P <* .001) (mean difference: –1.07; 95% CI, –1.7 to –0.48) compared with the NH4-Ac alone–stimulated mice (n = 9) and there was no difference compared with control mice (n = 12). (*B*) Administration of ODN2088, along with NH_4_-Ac prevented the rise in the liver-to-body weight ratio in WT mice (n = 15) (*P <* .0001) (mean difference: –0.02; 95% CI, –0.026 to –0.014) compared with the NH4-Ac alone–stimulated mice (n = 16) and there was no difference compared with control mice (n = 13). (*C*) Following NH_4_-Ac stimulation, there was a significant increase in the hepatocyte swelling in the liver histology (hematoxylin and eosin stain, 200× magnifications) in WT mice compared with control mice, where the cytoplasm of cells remained intact. Administration of ODN2088, ameliorated the hepatocyte swelling in WT mice. The images in the black box inset are the representative images at 400× magnification. (*D*) Following NH_4_-Ac stimulation, there was a significant increase in the total plasma DNA level in WT mice (n = 12) (*P =* .02) (mean difference: 12.58; 95% CI, 0.5 to 5.4) and in WT mice administered with ODN2088 (n = 16) (mean difference: 11.4; 95% CI, 0.6 to 5.2) compared with control mice (n = 12). There was no difference in the total plasma DNA level in NH_4_-Ac alone WT mice stimulated compared with ODN2088 treated group. (*E*) Administration of ODN2088, along with NH_4_-Ac prevented the rise in the intracellular cytokine IFNγ produced by splenic macrophages in WT mice (n = 11) (*P <* .0001) (mean difference: –2.5; 95% CI, –3.1 to –1.9) compared with the NH4-Ac alone–stimulated mice (n = 8) and there was no difference compared with control mice (n = 7). (*F*) Administration of ODN2088, along with NH_4_-Ac prevented the rise in the intracellular cytokine TNFα produced by splenic macrophages in WT mice (n = 11) (*P <* .0001) (mean difference: –28.9; 95% CI, –32.1 to –25.6) compared with the NH4-Ac alone–stimulated mice (n = 8) and there was no difference compared with control mice (n = 7). (*G*) Administration of ODN2088, along with NH_4_-Ac prevented the rise in the intracellular cytokine IL-6 produced by splenic macrophages in WT mice (n = 11) (*P <* .0001) (mean difference: –5; 95% CI, –5.5 to –4.5) compared with the NH4-Ac alone–stimulated mice (n = 8) and there was no difference compared with control mice (n = 7). (*H*) Representative FACS plots of the intracellular cytokines IFNγ, TNFα, and IL-6 produced by splenic macrophages in WT control mice and NH_4_-Ac–stimulated WT mice with and without ODN2088.

**Figure 10:**
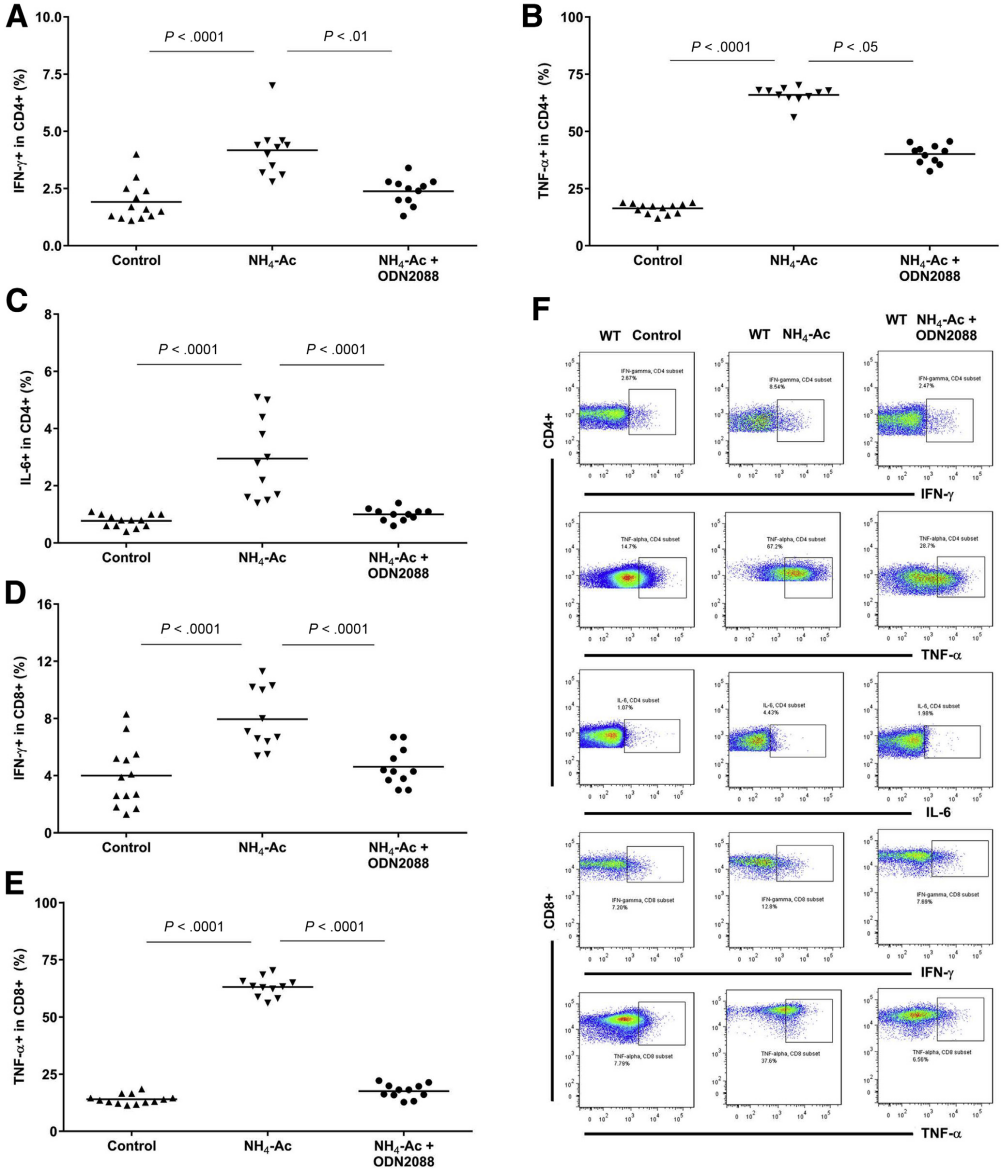
**ODN2088 inhibits the ammonia-induced cytokine production by T cells in WT mice.** Administration of the TLR9 antagonist, ODN2088, along with NH_4_-Ac significantly inhibited (*A*) the intracellular cytokine IFNγ (*P <* .01) (median difference: –1.8; 95% CI, –2.7 to –0.9), (*B*) the intracellular cytokine TNFα (*P <* .05) (median difference: –26; 95% CI, –29.5 to –22), and (*C*) the intracellular cytokine IL-6 (*P <* .001) (mean difference: –2; 95% CI, –2.8 to –1.1) produced by splenic CD4^+^ T cells in WT mice (n = 11) compared with the NH4-Ac alone–stimulated WT mice (n = 11). ODN2088 along with NH_4_-Ac also significantly inhibited (*D*) the intracellular cytokine IFNγ (*P <* .0001) (mean difference: –3.3; 95% CI, –5.4 to –1.3) and (*E*) the intracellular cytokine TNFα (*P <* .0001) (mean difference: –45.5; 95% CI, –49 to –42) produced by splenic CD8^+^ T cells in WT mice (n = 11) compared with the NH4-Ac alone–stimulated WT mice (n = 11). (*F*) Representative FACS plots of the intracellular cytokines IFNγ, TNFα, and IL-6 produced by splenic T cells in WT control mice and NH_4_-Ac–stimulated WT mice with and without ODN2088.

As *Tlr9*^*–/–*^ mice were protected against NH_4_-Ac stimulation, we tested whether an antagonist of TLR9 (ODN2088) could inhibit the ammonia-induced proinflammatory changes and brain edema observed in the WT mice. Administration of ODN2088 (50 μg/mouse) with NH_4_-Ac (4 mM) significantly decreased the BW content and liver-to-body weight ratio and ameliorated the hepatocyte swelling (Figure 9*A–C*). Administration of ODN2088 did not alter the total plasma DNA levels in WT mice following NH_4_-Ac stimulation but they were increased compared with control mice (Figure 9*D*). There was also a significant reduction in the cytokines produced by macrophages (Figures 9*E–G*) and T cells (Figure 10*A–F*).

## Discussion

In this study, we have demonstrated a novel link between ammonia-induced inflammation and the subsequent development of brain edema mediated by TLR9 using different mouse models. In addition, we showed that TLR9 expressed by lysozyme-producing cells in mice was critical for ammonia-induced brain edema and inflammation to develop. Importantly, we also showed that administration of a TLR9 antagonist abrogated the inflammation and prevented brain edema supporting therapeutic exploration of TLR9 antagonism in early ALF.

Ammonia stimulation upregulated the production of the cytokines IFNγ, TNFα, and IL-6 by macrophages and T cells. The observation of increased cytokine production from the hepatic-infiltrated T cells indicates that the immune response originates from the liver. The cytokines IFNγ, TNFα, and IL-6 produced by T cells are predominantly responsible for mediating inflammation within the liver.^21^, ^22^, ^23^ In this study ammonia activated cytokine production in macrophages indicated that those cells, along with CD4^+^ and CD8^+^ T cells, are responsible for promoting the proinflammatory environment. It is possible that ammonia and systemic inflammation induce brain edema by inducing astrocyte swelling. This is supported by the previously published observation that astrocyte swelling can be induced when astrocytes are exposed to proinflammatory cytokines after being exposed to ammonia.^11^ Though deficiency of TLR9 exacerbates cognitive impairment and severity of seizures in the brain,^24^ it has been demonstrated that activation of TLR9 in microglia and astrocytes induces the production of various proinflammatory cytokines in response to CpG DNA^25^ and activation of TLR9 in astrocytes leads to synaptic protein loss in the brain in chronic hyperglycemia.^26^ Activation of *Tlr9* results in the upregulation of cytokine production and increase in the BW content, and the critical role of TLR9 in this mechanism has been demonstrated by using genetically modified mice. TLR9 is essential for macrophage production of TNFα and IL-6 in response to CpG DNA and IFNγ produced by CD4^+^ T cells in response to CpG DNA and mycobacteria.^15^, ^27^ The observation that ammonia-induced TLR9 activation induces systemic inflammation and brain edema is well supported by recently published studies that show TLR9 induces inflammation in acetaminophen-induced ALF and nonalcoholic steatohepatitis.^16^, ^20^

Ammonia stimulation increased the total plasma DNA levels in all the different mouse models, thereby suggesting that ammonia activates TLR9 through DNA release. In acetaminophen-induced ALF, DNA fragments released by apoptotic hepatocytes have been shown to be responsible for the activation of TLR9 and induction of systemic inflammation.^16^, ^28^ These findings support the results of our recent human study, which showed profound TLR9 activation in neutrophils in the presence of plasma DNA in patients with acetaminophen-induced ALF, systemic inflammation, and brain edema,^17^ suggesting that TLR9 mediates the ammonia-induced brain edema in a DNA-driven manner. In this study, it is not possible to identify the source of the DNA, and we can only speculate that nonimmune cells are the source of DNA.

TLR9 expressed in the lysozyme-producing cells played an important role in the ammonia-induced cytokine production of macrophages and brain edema. Marques et al^29^ recently demonstrated that neutrophils are the predominant innate immune cells that sense DNA through the TLR9/nuclear factor-kappa B pathway and induce inflammation in the context of acetaminophen toxicity. These data are also well supported by the strong correlation demonstrated between neutrophil TLR9 expression, ammonia and IL-8 in acetaminophen-induced ALF and the abrogation of neutrophil TLR9 upregulation and cytokine production in acetaminophen-induced ALF plasma by DNAse-I.^17^

Administration of the TLR9 antagonist, ODN2088 abrogated the cytokine production and prevented the increase in BW content induced by stimulation of ammonia. These data are well supported by the findings of Imaeda et al,^16^ who in an acetaminophen-induced hepatotoxicity model, established that inhibition of TLR9 using ODN2088 and IRS954, a TLR7/9 antagonist, downregulated proinflammatory cytokine release and reduced mortality. The amelioration of brain edema and cytokine production by ODN2088 supports exploration of TLR9 antagonism as a therapeutic modality in early ALF to prevent the development of brain edema and intracranial hypertension.

There are pros and cons of using an ammonia-induced murine model of cerebral edema without acute liver injury. Some of the best-characterized animal models of cerebral edema in ALF include the hepatic devascularized rat^30^ and pig,^31^ and the rat with thioacetamide-induced toxic liver injury.^30^ While these models develop cerebral edema and exhibit intracranial hypertension, the impact of hepatic devascularization and necrosis bring other sequelae, including systemic inflammation, bacterial and fungal sepsis, and coagulopathy. This makes it hard to tease out the specific mechanisms that underpin the development of ammonia-induced brain edema per se and how this relates to innate immune dysfunction. Furthermore, we know that murine acetaminophen-induced ALF is mediated by TLR9 and can be abrogated by a TLR9 antagonist.^16^ Therefore, we could not utilize an acetaminophen-induced model of ALF to assess TLR9-mediated cerebral edema in this study and thus chose to examine this in a model of ammonia-induced cerebral edema.

In summary, this study demonstrates that the development of ammonia-induced brain edema requires macrophage and T cell expression of TLR9, which may be stimulated by DNA release. The importance of TLR9-expressing neutrophils or Kupffer cells in the induction of brain edema and inflammation was confirmed by the deletion of the TLR9 gene only in lysozyme-expressing cells. We have therefore demonstrated that TLR9 is necessary for the development of ammonia-induced brain edema.^16^, ^29^ The observation that systemic inflammation and brain edema can be prevented following administration of a TLR9 antagonist supports the exploration of TLR9 antagonism as a therapeutic modality in early ALF to prevent the progression to intracranial hypertension.

## Materials and Methods

### Animals Used for This Study

All the procedures and protocols used in the studies with animals were approved by the Institutional Animal Care and Use Committee at Yale University. Four strains of mice on C57BL/6 genetic background were used for the experiments. C57BL/6 (WT) were originally obtained from the Jackson Laboratory (Bar Harbor, ME). The original *Tlr9*^*–/–*^ and *Tlr9*^*fl/fl*^ breeders were kindly provided by Professor Shizuo Akira (Japan)^15^ and Professor Mark Shlomchik (University of Pittsburgh),^20^ respectively. We bred *Lysm-Cre* mice with *Tlr9*^*fl/fl*^ mice to generate *Lysm-Cre Tlr9*^*fl/fl*^ mice with TLR9 specific deletion in lysozyme expressing cells.^20^ All the mice used in the study were bred and kept at the Yale animal facility in specific pathogen–free conditions with autoclaved food, bedding, and filtered cage. The mice were fed on a regular chow diet and on a 12-hour light/dark cycle. Male mice (7–9 weeks old) were used in the study.

### Stimulation With Ammonium Acetate

To determine whether TLR9 plays a role in brain edema in the mice, NH_4_-Ac (NH_4_CH_3_CO_2_) (4 mmol/kg of bodyweight) was injected intraperitoneally in WT mice, *Tlr9*^*–/–*^ mice, *Lysm-Cre Tlr9*^*fl/fl*^ mice, and *Tlr9*^*fl/fl*^ mice that were sacrificed 6 hours after the injection. The concentration and duration of NH_4_-Ac used for this experiment was optimized in a pilot experiment. Na-Ac (NaCH_3_CO_2_) (J.T. Baker) (4 mmol/kg of bodyweight) was injected intraperitoneally in WT mice to demonstrate that any effect was due to ammonia and not the acetate moiety.

### Blood Collection and Tissue Harvesting

Six hours after the NH_4_-Ac stimulation, blood was collected from the mice and liver, spleen and brain were harvested. Plasma was collected and stored in –80°C. Spleen was homogenized using rough sides of 2 grinding slides and red blood cells were lysed by hypotonic solution and quickly restored in isotonic phosphate-buffered saline. The single suspension of splenocytes (10^6^) was stained with monocloncal antibodies (mAbs) conjugated with different fluorochromes before flow cytometry analysis. Liver tissue was homogenized using a plunge through a wire mesh and digested using collagenase-I and DNAse-I (Sigma-Aldrich, St Louis, MO). Liver-infiltrated immune cells were isolated from the homogenized tissue using the density-gradient Polymorphprep solution (Axis Shield, Oslo, Norway), stained with mAbs and analyzed using flow cytometry. The entire brain was weighed immediately after sacrifice using an electronic balance to determine the wet weight. The brain was then dried in an oven at 100°C for 24 hours to obtain the dry weight. The BW content was then calculated according to the formula^32^:

$$\text{BW content (\%) }=\;\frac{\text{Wet weight }-\text{ Dry weight}}{\text{Wet weight}}\text{× }100$$

### Preservation of Tissues for Histopathological Examination

For histopathological examination, tissues were harvested and stored in 10% formalin at room temperature. Specimens were then embedded in paraffin. Then, 6-μM tissue sections were stained with hematoxylin and eosin and examined under a light microscope.

### Total DNA Estimation

Total DNA was measured in the plasma samples stored at –80°C using the Quant-iT PicoGreen dsDNA quantitation kit (Life Technologies, UK) as detailed previously.^20^

### TLR9 Antagonist Injection

To determine whether an inhibitor of TLR9 offers protection against NH_4_-Ac stimulation, the TLR9 antagonist (ODN2088) (InvivoGen, San Diego, CA) (50 μg/mouse) was injected intraperitoneally in WT mice immediately following NH_4_-Ac injection. Six hours later, blood was collected and organs were harvested as mentioned above. The time and concentration of ODN2088 were chosen based on a recently established mouse model of acetaminophen hepatotoxicity.^16^

### Stimulation of Intracellular Cytokine Production

To determine the intracellular cytokine production of mononuclear cells from spleen and liver, up to 5.0 × 10^6^ cells per mL were stimulated with phorbol 12-myristate 13-acetate (50 ng) and ionomycin (InvivoGen, San Diego, CA) (500 pg) in complete media with Golgi plug (BD Biosciences, San Jose, CA) (1 μL) and incubated at 37°C for 5 hours in the presence of 5% CO_2_. At the end of 5 hours, stimulated cells were washed, stained with fluorochrome conjugated mAbs and analyzed using flow cytometry.

### Cell Staining and Flow Cytometry

Up to 1 million (1 × 10^6^) cells were resuspended in 100 μL of staining buffer and stained with different fluorochrome conjugated mAbs in a tube followed by incubation at room temperature in darkness for 30 minutes and the stained cells were washed with phosphate-buffered saline. For intracellular cytokine staining, 100 μL of cytofix/cytoperm solution (BD Biosciences, San Jose, CA) was added to the cell pellet after staining with surface markers and kept at room temperature for 20 minutes. The cells were washed with 1 mL of permeabilization wash buffer (BD Biosciences) followed by resuspension in 300 μL of phosphate-buffered saline and acquired in a LSRII flow cytometry (BD Biosciences) using BD FACS DIVA software V6.0 (BD Biosciences).

### Neutrophil Phagocytosis

The phagocytic ability of neutrophils was determined by incubating the whole blood with Dextran FITC (1 mg/mL) at 37°C for 20 minutes in a water bath and measuring the mean fluorescence intensity of the neutrophils in a flow cytometer. Neutrophils were identified using Lys6-G (1A8) and CD11b (M1/70) antibodies.

### Identification and Characterization of Various Immune Cell Subsets

The different subsets of lymphocytes were identified using their specific markers. T cells were identified using CD3 (17A2); CD4 (GK1.5) and CD8 (53-6.7) markers were used to characterize the different subsets of T cells and macrophages were identified using F4/80 (BM8) and CD11b (M1/70) markers. Intracellular cytokine production (IL-6 [MP5-20F3], IFNγ [XMG1.2], and TNFα [MP6-XT22]) was determined in the CD4^+^ and CD8^+^ T cell subsets and F4/80^+^ and CD11b^+^ macrophages. Flow cytometry antibodies were purchased from BioLegend (San Diego, CA).

### Statistics

For comparisons between 2 groups, Student’s *t* test (parametric data) or Mann-Whitney *U* test (nonparametric data) were used; for comparisons among 3 or more groups, 1-way analysis of variance with Tukey’s multiple comparison tests (parametric data) or Kruskal Wallis with Dunn’s multiple comparison tests (nonparametric data) were used based on the normal distribution of the data. All the results are presented as mean or median differences with 95% confidence intervals. Hypothesis testing was 2 tailed at an alpha level of .05. All statistical analyses were performed using GraphPad Prism 7.0 (GraphPad Software, San Diego, CA); *P* < .05 was considered as statistically significant.

## References

[1] Bernal W., Hall C., Karvellas C.J., Auzinger G., Sizer E., Wendon J. (2007). Arterial ammonia and clinical risk factors for encephalopathy and intracranial hypertension in acute liver failure. Hepatology.

[2] Phear E., Sherlock S., Summerskill W. (1955). Blood ammonia levels in liver disease and hepatic coma. Lancet.

[3] Butterworth R.F. (2015). Pathogenesis of Hepatic Encephalopathy and Brain Edema in Acute Liver Failure. Journal of Clinical and Experimental Hepatology.

[4] Vaquero J., Polson J., Chung C., Helenowski I., Schiodt F.V., Reisch J., Lee W.M., Blei A.T. (2003). Infection and the progression of hepatic encephalopathy in acute liver failure. Gastroenterology.

[5] Rolando N., Wade J., Davalos M., Wendon J., Philpott-Howard J., Williams R. (2000). The systemic inflammatory response syndrome in acute liver failure. Hepatology.

[6] Wright G., Shawcross D., Olde Damink S.W.M., Jalan R. (2007). Brain cytokine flux in acute liver failure and its relationship with intracranial hypertension. Metab Brain Dis.

[7] Jalan R., Damink S.W.O., Hayes P.C., Deutz N.E., Lee A. (2004). Pathogenesis of intracranial hypertension in acute liver failure: inflammation, ammonia and cerebral blood flow. J Hepatol.

[8] Blazka M.E., Wilmer J.L., Holladay S.D., Wilson R.E., Luster M.I. (1995). Role of Proinflammatory Cytokines in Acetaminophen Hepatotoxicity. Toxicol Appl Pharmacol.

[9] Ishida Y., Kondo T., Ohshima T., Fujiwara H., Iwakura Y., Mukaida N. (2002). A pivotal involvement of IFNγ in the pathogenesis of acetaminophen-induced acute liver injury. FASEB J.

[10] Dambach D.M., Watson L.M., Gray K.R., Durham S.K., Laskin D.L. (2002). Role of CCR2 in macrophage migration into the liver during acetaminophen-induced hepatotoxicity in the mouse. Hepatology.

[11] Rama Rao K.V., Jayakumar A.R., Tong X., Alvarez V.M., Norenberg M.D. (2010). Marked potentiation of cell swelling by cytokines in ammonia-sensitized cultured astrocytes. J Neuroinflammation.

[12] Kato M., Hughes R.D., Keays R.T., Williams R. (1992). Electron microscopic study of brain capillaries in cerebral edema from fulminant hepatic failure. Hepatology.

[13] Shawcross D.L., Jalan R. (2005). The pathophysiologic basis of hepatic encephalopathy: central role for ammonia and inflammation. Cell Mol Life Sci.

[14] Ratnakumari L., Qureshi I.A., Butterworth R.F. (1992). Effects of congenital hyperammonemia on the cerebral and hepatic levels of the intermediates of energy metabolism in spf mice. Biochem Biophys Res Commun.

[15] Hemmi H., Takeuchi O., Kawai T., Kaisho T., Sato S., Sanjo H., Matsumoto M., Hoshino K., Wagner H., Takeda K., Akira S. (2000). A Toll-like receptor recognizes bacterial DNA. Nature.

[16] Imaeda A.B., Watanabe A., Sohail M.A., Mahmood S., Mohamadnejad M., Sutterwala F.S., Flavell R.A., Mehal W.Z. (2009). Acetaminophen-induced hepatotoxicity in mice is dependent on Tlr9 and the Nalp3 inflammasome. J Clin Invest.

[17] Manakkat Vijay G.K., Ryan J.M., Abeles R.D., Ramage S., Patel V., Bernsmeier C., Riva A., McPhail M.J., Tranah T.H., Markwick L.J., Taylor N.J., Bernal W., Auzinger G., Willars C., Chokshi S., Wendon J.A., Ma Y., Shawcross D.L. (2016). Neutrophil Toll-like receptor 9 expression and the systemic inflammatory response in acetaminophen-induced acute liver failure. Crit Care Med.

[18] Kendrick S.F., O'Boyle G., Mann J., Zeybel M., Palmer J., Jones D.E., Day C.P. (2010). Acetate, the key modulator of inflammatory responses in acute alcoholic hepatitis. Hepatology.

[19] Warren K.S., Nathan D.G. (1958). The Passage of Ammonia Across the Blood-Brain-Barrier and its Relation to Blood pH. J Clin Invest.

[20] Garcia-Martinez I., Santoro N., Chen Y., Hoque R., Ouyang X., Caprio S., Shlomchik M.J., Coffman R.L., Candia A., Mehal W.Z. (2016). Hepatocyte mitochondrial DNA drives nonalcoholic steatohepatitis by activation of TLR9. J Clin Invest.

[21] Lin F., Taylor N.J., Su H., Huang X., Hussain M.J., Abeles R.D., Blackmore L., Zhou Y., Ikbal M.M., Heaton N., Jassem W., Shawcross D.L., Vergani D., Ma Y. (2013). Alcohol dehydrogenase-specific T-cell responses are associated with alcohol consumption in patients with alcohol-related cirrhosis. Hepatology.

[22] Blackmore L.J., Ryan J.M., Huang X., Hussain M., Triantafyllou E., Vergis N., Vijay G.M., Antoniades C.G., Thursz M.R., Jassem W., Vergani D., Shawcross D.L., Ma Y. (2015). Acute alcoholic hepatitis and cellular Th1 immune responses to alcohol dehydrogenase. Lancet.

[23] Yuksel M., Wang Y., Tai N., Peng J., Guo J., Beland K., Lapierre P., David C., Alvarez F., Colle I., Yan H., Mieli-Vergani G., Vergani D., Ma Y., Wen L. (2015). A novel "humanized mouse" model for autoimmune hepatitis and the association of gut microbiota with liver inflammation. Hepatology.

[24] Matsuda T., Murao N., Katano Y., Juliandi B., Kohyama J., Akira S., Kawai T., Nakashima K. (2015). TLR9 signalling in microglia attenuates seizure-induced aberrant neurogenesis in the adult hippocampus. Nat Commun.

[25] Butchi N.B., Du M., Peterson K.E. (2010). Interactions between TLR7 and TLR9 agonists and receptors regulate innate immune responses by astrocytes and microglia. Glia.

[26] Zhao Y., Pu D., Sun Y., Chen J., Luo C., Wang M., Zhou J., Lv A., Zhu S., Liao Z., Zhao K., Xiao Q. (2018). High glucose-induced defective thrombospondin–1 release from astrocytes via TLR9 activation contributes to the synaptic protein loss. Exp Cell Res.

[27] Bafica A., Scanga C.A., Feng C.G., Leifer C., Cheever A., Sher A. (2005). TLR9 regulates Th1 responses and cooperates with TLR2 in mediating optimal resistance to Mycobacterium tuberculosis. J Exp Med.

[28] Marques P.E., Amaral S.S., Pires D.A., Nogueira L.L., Soriani F.M., Lima B.H.F., Lopes G.A.O., Russo R.C., Ávila T.V., Melgaço J.G., Oliveira A.G., Pinto M.A., Lima C.X., De Paula A.M., Cara D.C., Leite M.F., Teixeira M.M., Menezes G.B. (2012). Chemokines and mitochondrial products activate neutrophils to amplify organ injury during mouse acute liver failure. Hepatology.

[29] Marques P.E., Oliveira A.G., Pereira R.V., David B.A., Gomides L.F., Saraiva A.M., Pires D.A., Novaes J.T., Patricio D.O., Cisalpino D., Menezes-Garcia Z., Leevy W.M., Chapman S.E., Mahecha G., Marques R.E., Guabiraba R., Martins V.P., Souza D.G., Mansur D.S., Teixeira M.M., Leite M.F., Menezes G.B. (2015). Hepatic DNA deposition drives drug-induced liver injury and inflammation in mice. Hepatology.

[30] Butterworth R.F., Norenberg M.D., Felipo V., Ferenci P., Albrecht J., Blei A.T. (2009). Experimental models of hepatic encephalopathy: ISHEN guidelines. Liver Int.

[31] Ytrebo L.M., Nedredal G.I., Langbakk B., Revhaug A. (2002). An experimental large animal model for the assessment of bioartificial liver support systems in fulminant hepatic failure. Scand J Gastroenterol.

[32] Tang J., Liu J., Zhou C., Alexander J.S., Nanda A., Granger D.N., Zhang J.H. (2004). Mmp-9 deficiency enhances collagenase-induced intracerebral hemorrhage and brain injury in mutant mice. J Cereb Blood Flow Metab.

